# Evaluating Artificial Intelligence in Full-Arch CBCT Caries Detection: A Comparative Analysis with Clinical Assessment

**DOI:** 10.3390/jcm15103841

**Published:** 2026-05-16

**Authors:** Jakub Kwiatek, Marta Leśna, Rafał Przybylski, Justyna Kaczewiak, Izabela Foryszewska, Sylwia Pokorska, Ilona Różewicz, Paulina Łojewska-Pabiś

**Affiliations:** 1 Kwiatek Dental Clinic, Kordeckiego 22, 60-144 Poznań, Poland; 2 Military Specialist Healthcare Center Oral and Maxillofacial Surgery Department, ul. Szylinga 1, 61-787 Poznań, Poland

**Keywords:** dental caries, dental diagnostics, artificial intelligence (AI), diagnocat, machine learning, cone beam computed tomography (CBCT), blind study

## Abstract

**Objectives:** The aim of this study was to compare the diagnostic accuracy of the Diagnocat system (DGNCT LLC, Miami, Florida, USA), based on artificial intelligence algorithms, with clinical assessments performed by three dentists. **Materials and Methods:** The analysis was based on data obtained from cone-beam computed tomography (CBCT), focusing on the detection of carious lesions. The inclusion of three specialists with comparable levels of knowledge and professional experience increased the reliability of the results. The dentists classified teeth with carious lesions solely on the basis of CBCT imaging, physical examination, and their own clinical knowledge, under single-blind conditions, without awareness of the subsequent comparative analysis. **Results:** The results demonstrated a variable level of agreement between the Diagnocat system and the dentists’ assessments, depending on factors such as tooth location, as well as patient age and gender. The lowest level of agreement was observed in premolars, which may be attributed to their complex morphology. Higher diagnostic accuracy was noted in molars and incisors, particularly in younger patients. **Conclusions:** Further research should focus on the integration of various diagnostic modalities, including diagnostic imaging, intraoral scans, and photographic documentation, which may significantly enhance diagnostic precision, especially in cases of early-stage lesions. According to the results, the Diagnocat system demonstrates potential as a supportive tool in the diagnostic process in dental practice, as well as a screening tool enabling preliminary evaluation of imaging studies.

## 1. Introduction

Artificial intelligence (AI), and in particular convolutional neural networks (CNNs), due to their ability to efficiently and repeatedly detect pathological changes, are revolutionizing the analysis of imaging studies across numerous fields of medicine [1].

It has been demonstrated that their use in the evaluation of screening examinations increases diagnostic accuracy in the early detection of breast cancer [2], enables the identification of skin cancers at a level comparable to dermatologists [3], as well as the detection of pneumonia on radiographic images [4] and diabetic retinopathy [5].

The implementation of artificial intelligence in dental diagnostics enhances efficiency and improves treatment effectiveness, thereby optimizing clinical workflows. Advances in this field, due to the vast potential for reliable and objective diagnostics, have significantly improved diagnostic accuracy, particularly in complex cases such as periodontal diseases, tooth fractures, oral infections, and the detection of dental caries. AI algorithms have demonstrated the ability to detect changes in bone density in imaging studies with accuracy often exceeding human capabilities, which is of importance in the early detection of periodontal diseases and in implant treatment planning. However, this requires further research focused on integrating AI with various imaging modalities and providing more heterogeneous imaging datasets—from different devices and from patients of diverse ethnic backgrounds [6,7,8].

Given that most studies discussed in the literature are conducted under isolated conditions—without patient involvement—the implementation of AI models into routine dental practice will require collaboration between clinicians and software developers, the establishment of standardized protocols [9] as well as further clinical studies to validate findings and address the limitations of previous research [7,10,11]. It is anticipated that the future benefits of artificial intelligence in dental practice will lead to more personalized treatment plans and improved patient outcomes.

Imaging studies play a crucial role in contemporary dentistry. Intraoral radiographs and orthopantomograms have long been essential diagnostic tools; however, they are associated with limitations inherent to two-dimensional (2D) imaging. The advent of cone beam computed tomography (CBCT) has enabled greater diagnostic precision and accuracy in dentistry by providing high-resolution three-dimensional cross-sectional images [12]. CBCT devices emit an X-ray beam in a cone-shaped configuration, as opposed to the fan-shaped beam used in conventional computed tomography scanners, resulting in significantly lower radiation doses. Conventional computed tomography still provides superior image quality, particularly when information regarding soft tissues is required, and allows for the use of intravenously administered contrast agents, which is especially important in cases of suspected head and neck malignancies. However, for routine use in daily clinical practice, CBCT is justified due to its lower radiation dose and the greater availability of CBCT devices in dental offices [13]. An undeniable advantage of this method over standard 2D radiographs is the possibility of reconstruction, as well as the absence of distortion and superimposition of anatomical structures [14,15]. Over the years, an increasing trend among dentists toward more frequent use of CBCT in daily clinical practice has been observed [16], including in the assessment of temporomandibular joints [17], planning of orthognathic surgeries [18], dental implant placement [19], maxillary sinus floor elevation procedures [20] and endodontics [21], thereby enhancing its significance as a diagnostic tool. A study conducted by Kumar N. et al. [22] demonstrated a systematic increase in CBCT utilization, particularly in implant planning—consistent with the guidelines of the American Academy of Oral and Maxillofacial Radiology, which recommend CBCT as the method of choice for imaging implant sites, as well as for the evaluation of impacted teeth and the diagnosis of pathological lesions. The authors emphasize that CBCT is becoming increasingly integrated into routine clinical practice, reflecting the growing trend toward the use of 3D imaging in dentistry [22,23].

Despite the higher radiation dose associated with CBCT compared to 2D imaging, continuous advancements in low-dose protocols and artifact reduction algorithms have expanded its clinical applications. Integration with digital tools, such as intraoral scanners, Computer-Aided Design (CAD) and Computer-Aided Manufacturing (CAM) systems, has further increased its utility in implant surgery and orthodontic appliance design [24]. However, it demonstrates significant clinical utility in cases where clinical symptoms do not correspond with findings from radiovisiographic (RVG), panoramic (OPG) images or when the image is unclear or ambiguous. Additionally, CBCT performed in the context of implant planning may simultaneously allow for the assessment of the presence of caries. It should be noted that the radiological appearance of caries may change significantly depending on the projection geometry [14,25].

Interpretation of CBCT examinations is time-consuming and largely dependent on the clinician’s experience. Studies have shown that the amount of training devoted to the interpretation of three-dimensional imaging among dentists is insufficient [26,27]. Therefore, support in the form of artificial intelligence–based tools is important for optimizing treatment [25]. Among 305 surveyed dentists, a substantial majority expressed willingness to refer CBCT scans for external review if such an option were available. Diagnostic confidence regarding CBCT interpretation was reported by only 31.5% of the participants [28].

The accuracy of CBCT analysis is further constrained by the specific clinical conditions under which diagnostics are performed. These include time constraints and the necessity to focus on the primary clinical indication. Consequently, there is a risk of oversight regarding incidental findings that fall outside the immediate area of diagnostic interest [29,30].

The implications of these limitations are particularly pronounced in the diagnosis of prevalent conditions frequently overlooked during three-dimensional imaging analysis, such as dental caries. To address these constraints, the methodology of the present study incorporates an artificial intelligence system operating within real-world clinical conditions—an approach that represents a novel element compared to prior investigations [30].

Oral diseases constitute a major public health problem on a global scale [31]. According to data presented in the literature, dental caries was the 10th most prevalent disease worldwide, affecting 2.4 billion people in 2015 [32]. It disproportionately affects populations of low socioeconomic status and, despite being largely preventable, its prevalence has remained stable over the past three decades [33].

Given the enormous potential of artificial intelligence in medicine to optimize workflow, improve treatment outcomes, and enable personalized treatment planning, there is a strong need for studies analyzing its use in clinical settings. In response to this need, and building on our previous analyses, we conducted a comparative study aimed at evaluating the effectiveness of the Diagnocat program in detecting dental caries on CBCT images, in comparison with clinical assessments performed by three general dentists without specialization, each with comparable professional experience of at least five years [30].

## 2. Material and Methods

### 2.1. Description of the Study Population

This retrospective cross-sectional study analyzed data from 100 patients who presented to the Kwiatek Dental Clinic. The study cohort comprised 54 females and 46 males, with ages ranging from 18 to 78 years. To ensure methodological consistency, the inclusion and exclusion criteria, the sample size, and the clinical evaluation protocol (performed by the same three general dentists) were designed to be identical to those of the prior study [30].

Patients were selected for this analysis after meeting the inclusion criteria:

- age over 18 years,
- first-time visit to the clinic with one of the 3 general dentists without specialization who had participated in the previous study,
- availability of diagnostically acceptable CBCT scans of the maxilla and mandible.

The exclusion criteria were:

- age below 18 years,
- pregnancy,
- absence of a dual-arch CBCT scan
- edentulism,
- insufficient quality of the examination.

Data were collected between May 2024 and July 2025.

### 2.2. Research Procedure

#### 2.2.1. Description of the AI Software (Diagnocat, DGNCT LLC, Miami, FL, USA)

**Figure 1:**
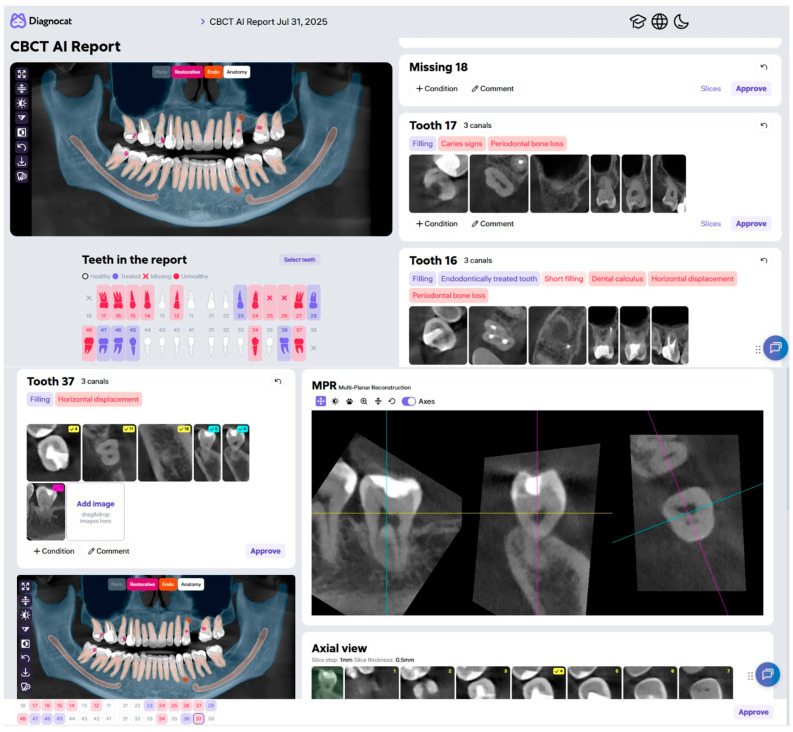
Screenshot of the AI Program “Diagnocat”—Report from CBCT analysis.

Diagnocat is a convolutional neural network (CNN)-based system designed to provide clinical decision support for precise dental diagnostics. It facilitates the analysis of diverse imaging modalities, including panoramic radiographs and CBCT scans, by automatically identifying pathologies such as dental caries, periapical lesions, and other abnormalities (Figure 1). Its capabilities undergo continuous enhancement through systematic refinement [34,35,36].

#### 2.2.2. Data Collection Process

The study was retrospective and had a cross-sectional design. Patient selection and recruitment were performed using ProDentis 9.99 software. Patients who had undergone CBCT scans of the maxilla and mandible using the Orthophos SL 3D device (Dentsply Sirona, Charlotte, NC, USA) were included.

Based on the clinical examination and the obtained CBCT, the dentist diagnosed the presence of caries. Primary caries is defined as a carious lesion developing on previously sound tooth surfaces that have not been undergoing restorative treatment. In contrast, secondary caries refers to lesions originating at the margins of, or beneath, existing dental restorations [37].

In the present analysis, all carious lesions were considered collectively as a single diagnostic category, regardless of whether they represented primary or secondary caries. This approach was consistent with the aim of the study, which was to assess the overall prevalence and diagnostic detection of caries rather than to analyze differences between lesion types or etiologies. Moreover, the AI software (Diagnocat, version 2.0) evaluated in this study does not currently distinguish between primary and secondary caries lesions; therefore, the same uniform classification was applied in the clinical assessment to ensure a direct and valid comparison between the automated and clinical diagnostic outcomes. This approach is also commonly used in epidemiological studies [38], where caries assessment primarily focuses on the presence and severity of lesions rather than their origin. Aggregating all carious lesions into a single category also allowed for more robust statistical analysis and reduced the issue of potentially small sample sizes within individual subgroups.

Following the clinical assessment, the identical CBCT datasets were processed and analyzed via the Diagnocat system. The diagnoses used in this study were established based on CBCT, as well as the clinical experience and medical knowledge of the dentists.

To ensure consistency of the analyses, the study included clinicians whose clinical assessments had been analyzed in a previous study [30].

The clinicians, as in the original study, were not informed that their diagnoses would be compared with the results of analyses performed using the Diagnocat system. This was intended to eliminate the potential influence of awareness of AI-assisted analysis on the diagnostic process and to reduce assessment bias.

#### 2.2.3. Statistical Analysis Methods

To evaluate the performance of the AI software in caries detection and its diagnostic agreement with the clinician’s assessment, a comparative analysis was performed.

Descriptive analysis involved the computation of means with standard deviations (SD), medians with interquartile ranges (IQR), and 95% confidence intervals (CI).

To compare results and assess agreement between Diagnocat analyses and clinical evaluations, the Pearson’s chi-square test (χ^2^) was applied. It enabled comparison of proportions across groups such as patient gender, age, and type of examined teeth. In addition, the test allowed for the detection of differences in assessments generated by the Diagnocat software depending on the dentists performing the evaluation.

Pairwise comparisons of proportions were conducted using the two-proportion Z-test to identify specific differences between the study groups.

Analysis of variance (ANOVA) was used to evaluate the agreement between Diagnocat and clinical assessments across tooth types and age groups. This statistical approach further enabled the identification of significant differences between the studied populations.

The level of statistical significance was determined based on *p*-values, with *p* < 0.05 considered the threshold for significance. The most important results of the analysis were presented in the form of tables and figures, with *p* < 0.05 values highlighted in bold. Results indicating a tendency toward statistical significance (0.05 ≤ *p* < 0.1) were additionally emphasized to indicate potential trends requiring further research.

### 2.3. Evaluation Criteria

#### Categories of Analysis: Gender, Age, Dentist

The overall detectability of carious lesions was assessed for each of the 32 potential permanent teeth, excluding those missing in individual patients. The analysis accounted for variables such as patient gender and age, as well as tooth groups (incisors, canines, premolars, and molars), tooth numbering (11–48), and the evaluating clinician.

Consistent with the methodology established in the previous study, variables such as gender age, and tooth group were incorporated into the analysis [30].

## 3. Results

**Figure 2:**
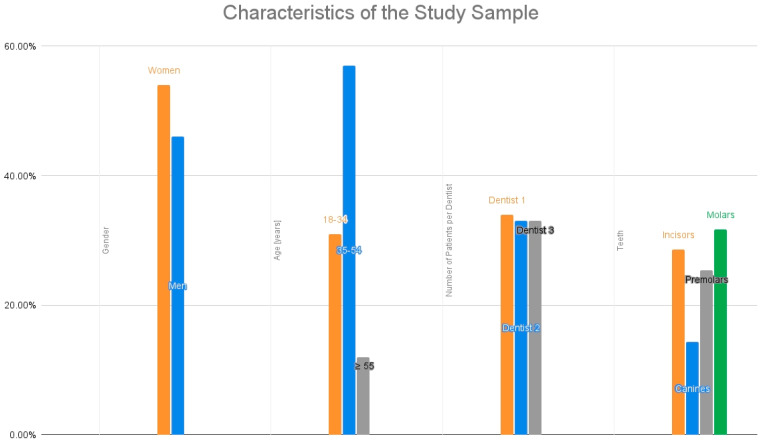
Characteristics of the study sample.

The overall demographic and clinical characteristics of the study population are presented in Table 1 and Figure 2. Data were extracted from patients’ medical records via retrospective chart review.

### 3.1. Overall Effectiveness of Diagnocat (DGNCT LLC, Miami, FL, USA)

The reference standard for this analysis was the diagnosis established through clinical examination. A total of 2755 teeth were evaluated across 100 patients; 445 teeth were identified as missing, including third molars.

Diagnocat detected 311 carious lesions, whereas dentists identified 487 lesions. The software incorrectly indicated the presence of caries in 64 teeth and failed to detect ongoing carious processes in 240 teeth (Table 2).

### 3.2. Analysis of the Total Number of Carious Lesions and Detection of Healthy Teeth

**Figure 3:**
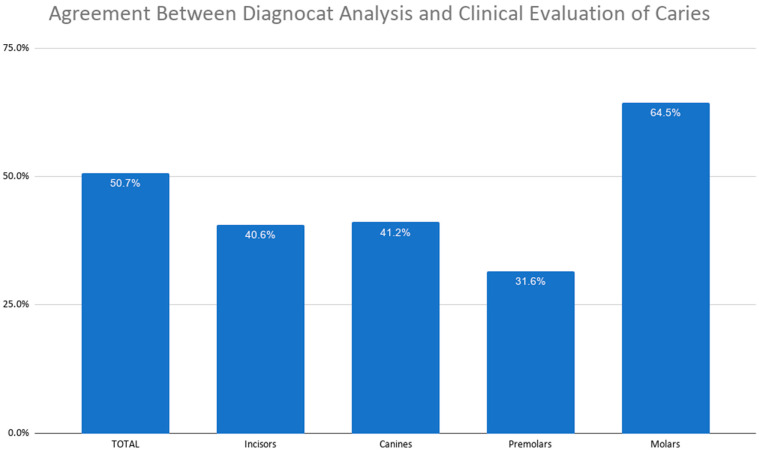
Agreement between Diagnocat analysis and clinical evaluation of caries.

The analysis focused on the occurrence of dental caries. The mean diagnostic agreement between the Diagnocat system and the clinicians’ CBCT-based assessments was 50.7%. The lowest level of agreement (31.6%) was observed for premolars, while the highest was recorded for molars (64.5%) (Figure 3).

**Figure 4:**
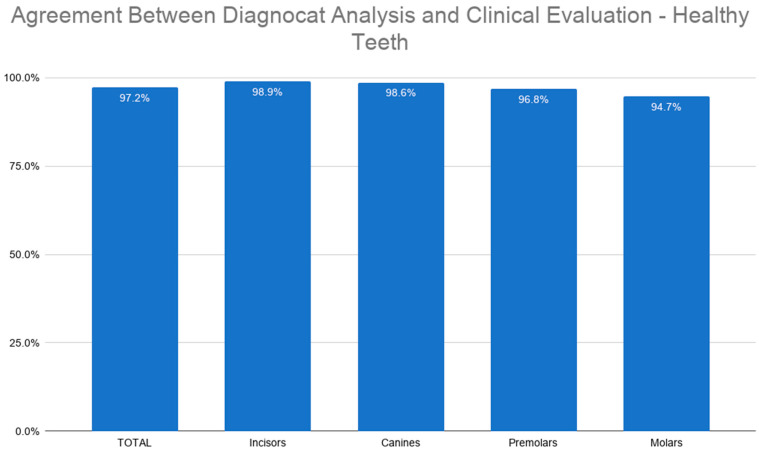
Agreement between Diagnocat analysis and clinical evaluation—healthy teeth.

Regarding the identification of sound teeth, the agreement between the AI and clinical assessment was 97.2%. The highest concordance was observed for incisors (98.9%), while the lowest was recorded for molars (94.7%). For canines and premolars, the agreement reached 98.6% and 96.8%, respectively (Figure 4).

In detecting carious lesions, the Diagnocat system demonstrated a sensitivity of 50.8%, a specificity of 97.2%, and an overall diagnostic accuracy of 89%.

#### 3.2.1. Agreement Between Diagnocat Analysis and Clinical Evaluation by Gender

**Figure 5:**
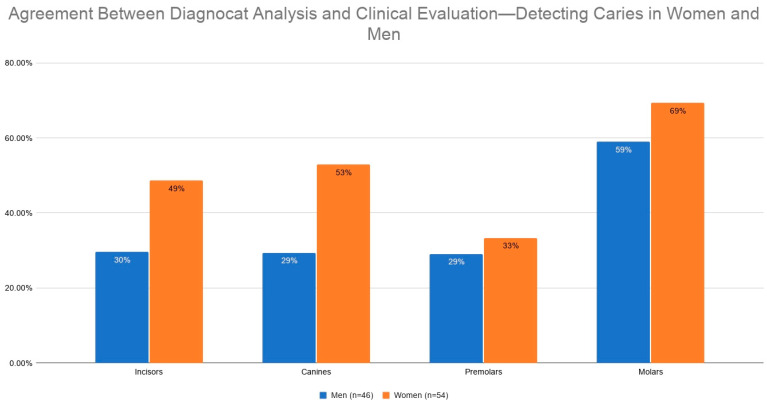
Agreement between Diagnocat analysis and clinical evaluation—detecting caries in women and men.

Differences in agreement between the Diagnocat system and clinical assessment were observed in relation to patient gender (Table 3, Figure 5).

This difference was statistically significant for all analyzed teeth combined (*p* = 0.0435, χ^2^ = 4.08), with higher agreement observed in women (54.89%) than in men (45.7%). The data showed a trend toward gender-related differences in the molar region (*p* = 0.0829, χ^2^ = 3.01), with agreement rates of 69.4% for women and 59.02% for men (Table 4).

#### 3.2.2. Agreement Between Diagnocat Analysis and Clinical Evaluation by Patient Age

Agreement results were analyzed across three age categories: 18–34 years (*n* = 31), 35–54 years (*n* = 57), and 55 years and older (*n* = 12). Consistent with our earlier methodology, these divisions reflect age-specific variations in caries risk, bio-physiological changes, and levels of oral hygiene and preventive care [30].

**Figure 6:**
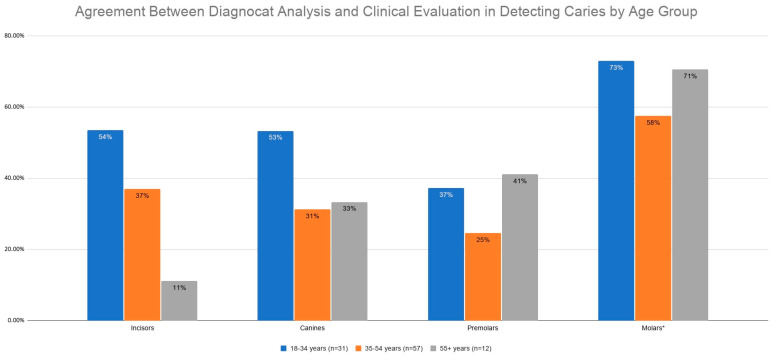
Agreement between Diagnocat analysis and clinical evaluation in detecting caries by age group. Note: The chi-square test of independence. * Statistically significant difference detected for this tooth type.

An analysis accounting for all teeth revealed a statistically significant difference in the agreement between the Diagnocat system and clinical assessment across different age categories (χ^2^ = 32.67, *p* = 0.0088). The Z-Test for proportions demonstrated a statistically significant difference between the youngest study group and the middle-aged group. Additionally, a trend was observed between the youngest and oldest age groups, with the highest level of agreement consistently occurring in the youngest age category (Table 5, Figure 6).

The agreement between the Diagnocat system and dental assessment differs significantly across the evaluated age categories and tooth types. The highest agreement was observed in the 18–34 age group (59.28%), whereas in the 35–54 and over 55 age groups it was very similar (44.94% and 45.65%, respectively). Pairwise comparisons using the two-proportion Z-test revealed statistically significant differences between the youngest and middle-aged groups (*p* = 0.0028). While the difference between the youngest and oldest groups did not reach the threshold for significance, a trend was observed (*p* = 0.0935).

In the case of incisors, the agreement was highest in the 18–34 group (53.57%) compared to the 35–54 group (37.04%) and the 55+ group (11.11%), and this difference was close to statistical significance (*p* = 0.0520). The two-proportion Z-test revealed a statistically significant difference between group 1 and group 3 (*p* = 0.0253).

For canines, the highest agreement was also observed in the 18–34 age group (53.33%). Agreement rates were lower in the middle-aged (31.25%) and oldest (33.33%) groups; however, these differences did not reach statistical significance.

Regarding premolars, agreement was highest in the oldest group (41.18%) and lowest in the middle-aged group (24.62%), with the youngest group reaching 37.25%. These differences were not statistically significant (*p* = 0.2279), and subsequent pairwise comparisons using the two-proportion Z-test confirmed the lack of significance between the groups.

For molars, the highest agreement was observed in the youngest group (73.0%), followed by the oldest (70.59%) and the middle-aged group (57.55%). These differences were statistically significant (*p* = 0.0401) with the two-proportion Z-test revealing a significant difference specifically between group 1 and group 2 (Table 6).

The agreement between the Diagnocat system and clinical assessment was further evaluated for individual teeth across the defined age cohorts (Table 7).

Statistically significant differences were identified for teeth 12 (*p* = 0.0428), 15 (*p* = 0.0490), 28 (*p* = 0.0396) and 35 (*p* = 0.0257). For teeth 12 and 28, the 18–34 age group achieved 100% agreement; in the 35–54 group, agreement rates were 25% and 70%, respectively, while the 55+ group showed 33.3% for both teeth. Similarly, for tooth 15, the highest agreement was observed in the youngest cohort (71.4%), followed by the middle-aged group (33.3%), whereas no agreement was noted in the oldest group. Conversely, for tooth 35, the oldest group (55+) reached 100% agreement, compared to 40% in the 35–54 group and 20% in the 18–34 group.

For teeth 21 (*p* = 0.0946), 45 (*p* = 0.0528), and 47 (*p* = 0.0669), the differences demonstrated a trend rather than reaching formal statistical significance. For tooth 21, the highest agreement was found in the youngest group (62.5%), with the middle-aged group at 33.3% and no agreement in the oldest cohort. Regarding tooth 45, agreement was exclusively observed in the 18–34 group (42.9%). Finally, for tooth 47, agreement was limited to two groups: the youngest, which showed the highest rate (83.3%), and the middle-aged group (35.7%).

#### 3.2.3. Agreement Between Diagnocat Analysis and Clinical Evaluation by Dentist

**Figure 7:**
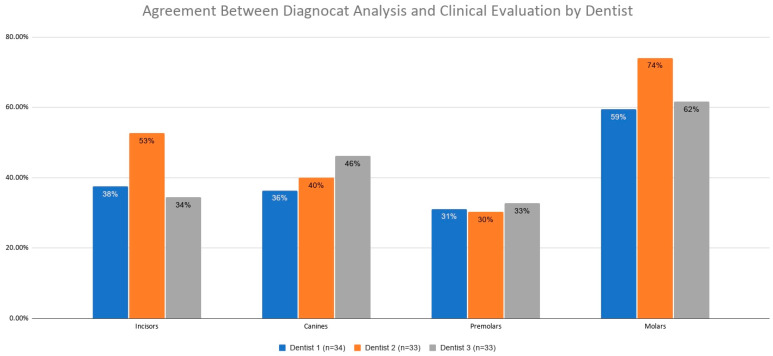
Agreement between Diagnocat analysis and clinical evaluation by dentist.

The agreement between the Diagnocat system and clinical assessment for caries detection was also stratified by the examining dentist (Figure 7, Table 8).

The analysis accounting for all teeth showed a trend toward differences between clinicians (χ^2^ = 5.71, *p* = 0.0981), with the highest agreement rate achieved by Clinician 2 (58.27%) (Table 9). Pairwise comparisons using the two-proportion Z-test revealed a statistically significant difference between Dentist 2 and Dentist 3 (*p* = 0.0389). Additionally, a trend was observed between Dentists 1 and 2 (*p* = 0.0965).

Subgroup analysis by tooth type revealed a statistically significant difference in the two-proportion Z-test for molars when comparing Dentists 1 and 2 (*p* = 0.0402).

A comparative analysis of clinical assessment was also performed, taking into account individual teeth (Table 10). A statistically significant difference was observed for tooth 15 (*p* = 0.0490), where Dentist 2 achieved 100% agreement, compared to 33.33% for Dentist 1 and 28.6% for Dentist 3.

A trend was observed for teeth 16, 23, and 47 (*p* = 0.0590, *p* = 0.0770, and *p* = 0.0835, respectively), although these values did not reach the threshold for statistical significance. Regarding tooth 16, Dentist 2 again achieved 100% agreement, followed by Dentist 1 (71.4%) and Dentist 3 (50.0%). For tooth 23, the highest agreement was demonstrated by Dentist 3 (75.0%) and Dentist 1 (50.0%), whereas Dentist 2 showed no agreement. For tooth 47, agreement rates were 80.0% for Dentist 3, 57.1% for Dentist 2, and 22.2% for Dentist 1.

### 3.3. Analysis of the Impact of Patient Group Structure (Age, Gender) on the Agreement Between Diagnocat and Doctors’ Evaluations

Statistical analysis was conducted to evaluate the relationships between the studied variables (clinician, age, and gender). The distribution of patients across the specified age cohorts (18–34, 35–54, and 55+ years) was comparable among the examining clinicians and showed no statistically significant differences (Table 11). Similarly, the analysis of the relationship between patient gender and the examining clinician revealed no significant differences, with a comparable distribution of male and female patients across all clinicians (Table 12).

## 4. Discussion

The results obtained from the CBCT analysis confirm previous findings based on 2D imaging highlighting the influence of patient age, gender, tooth type, and inter-examiner variability on diagnostic concordance. Despite the system’s high specificity, its relatively low sensitivity indicates a potential for significant false-negative rates. These findings suggest that the system may overlook a substantial number of pathologies, thereby limiting its clinical utility as a standalone diagnostic tool.

Current research indicates that artificial intelligence-based systems can achieve high diagnostic accuracy. Esmaeilyfard et al. [14] reported a diagnostic accuracy of approximately 95%, with high sensitivity and specificity for caries detection in molars. However, these superior results may be attributed to differences in study methodology, specifically, their analysis was conducted on isolated teeth rather than entire dental arches within CBCT examinations. Restricting the diagnostic field to isolated structures significantly reduces diagnostic complexity and the prevalence of imaging artifacts, which likely leads to inflated performance metrics compared to full-arch clinical scenarios.

In a study by Kaźmierczak et al. [39] which evaluated dentition at both the individual tooth and full-arch levels, the Diagnocat system assessed the presence or absence of missing teeth, restorations, endodontic treatments, bridge pontics, orthodontic appliances, crowns, and implants in CBCT scans. While the system achieved nearly perfect precision (over 99%) during the analysis of individual teeth, its performance significantly declined when conducting a comprehensive evaluation of the entire oral cavity.

Similarly, in a study by Ezhov et al. [15] the Diagnocat system was used to analyze full-volume CBCT scans, automatically segmenting anatomical structures and identifying pathologies. The authors demonstrated high system specificity and reported improved diagnostic performance among clinicians utilizing AI support. These findings confirm the potential value of such tools as supplement to the diagnostic process rather than full replacements for clinical judgment.

The analysis revealed differences in diagnostic agreement between the AI tool and clinical assessment based on patient gender. Specifically, a higher overall agreement rate for all teeth combined was observed in female patients.

While artificial intelligence has been extensively studied in CBCT-based diagnostics, current literature does not address potential gender-related differences in AI performance or its concordance with clinical evaluation. Consequently, a direct comparison of these results with existing data is currently not possible. Nevertheless, the presence of sexual dimorphism in odontometric parameters—such as crown dimensions and pulp chamber volume—may potentially influence detection efficacy. This underscores the need to consider gender as a factor in the training and validation processes of AI systems [40].

Our findings demonstrate a clear age-dependent trend in the diagnostic performance of the Diagnocat system, characterized by a general propensity for higher agreement within the youngest cohort. This pattern was particularly pronounced in the aggregate analysis of all teeth and specifically within the molar region, where consistency noticeably surpassed that of older generations. A compelling exception emerged in the premolar region, where the oldest population achieved the highest level of agreement; this may be attributed to more advanced pathological changes in older individuals, potentially presenting more distinct features that facilitate identification by the system.

Available literature does not directly address age-related differences in the diagnostic agreement of AI systems in CBCT imaging, precluding direct comparison. However, our findings suggest a distinct age-dependent pattern, particularly regarding tooth type. The notably higher variability observed in incisors among the oldest age group, compared to the youngest, stands in contrast to the stability of canine assessments, which remained consistent across all cohorts. Furthermore, the high level of agreement across specific teeth (such as 12, 15, 28, and 35) was most prominent in the youngest group.

This higher agreement observed in the younger population may be associated with age-dependent anatomical and structural factors, such as a lower prevalence of prosthetic restorations, fewer metallic artifacts, and reduced complexity of degenerative changes. However, these findings warrant further verification in studies involving larger and more diverse age cohorts.

Analysis revealed variations in agreement levels among clinicians. These findings highlight the presence of inter-observer variability, a well-documented phenomenon in radiology arising from differences in clinical experience, image interpretation techniques, and individual diagnostic strategies.

A notable strength of this study is the demographic homogeneity across the clinician groups. The lack of significant associations between clinician characteristics and patient demographics (age and gender) indicates that the study groups were well-balanced. This uniformity is crucial as it minimizes the risk of selection bias, ensuring that the observed differences in AI diagnostic agreement are likely attributable to inherent anatomical and structural factors rather than confounding variables in the study population.

This study extends previous 2D-based research by evaluating AI performance in the interpretation of full-arch CBCT scans, allowing for an assessment of diagnostic parameters within a three-dimensional imaging environment.

Our findings provide critical insights into the system’s efficacy across specific population subgroups, which carries significant clinical implications. Understanding these variations is important for identifying areas in which the algorithm may require further refinement and validation, particularly in patient groups or clinical conditions associated with lower diagnostic accuracy. At the same time, these findings emphasize that AI systems should currently be used primarily as decision-support tools, providing a valuable “second opinion,” rather than as autonomous diagnostic authorities.

This study has several limitations that should be considered when interpreting the results. First, the analysis was conducted on a relatively small patient sample within a single clinical center, which may limit the generalizability of the findings. Additionally, all CBCT scans were acquired using a single imaging device; this may have influenced the specific image characteristics and potentially limited comparability with scans obtained from other imaging systems.

Furthermore, the retrospective nature of the study and the lack of case randomization constitute additional limitations. The evaluation was also restricted to a single artificial intelligence platform, precluding a direct comparison of efficacy across different AI systems.

Finally, a significant limitation was the absence of a definitive ‘ground truth,’ such as a reference assessment performed by an expert panel, which could serve as the ultimate benchmark for evaluating diagnostic accuracy. Consequently, this limits the study to assessing concordance between the system and clinical evaluations rather than determining true diagnostic accuracy.

Future research should incorporate larger and more diverse patient populations, preferably within multicenter study designs, to enhance the reliability and generalizability of the findings.

Investigating gender-related differences in the performance of artificial intelligence systems should become a standard component of future investigations.

Furthermore, subsequent studies are required to determine whether the observed age-related variations reflect true biological variability, imbalances in training datasets, or inherent algorithmic bias.

Additionally, it is pertinent to compare different AI platforms used for CBCT image analysis and to conduct a more detailed assessment of technical factors, such as imaging artifacts and dental materials, which may impede algorithmic accuracy in radiographic interpretation.

A pivotal direction for future AI-driven caries detection models should be the integration of data from radiographic imaging, intraoral scans, and clinical photography. Combining these three modalities could potentially overcome current limitations in detecting early-stage demineralization and incipient caries, which often elude radiographic detection due to minimal hard-tissue loss.

Given the constraints of routine clinical practice—often characterized by time pressure and a focus on the patient’s chief complaint—future research should implement independent expert verification to establish a definitive ground truth. Conducting evaluations under conditions free from the demands of daily practice is essential for an objective assessment of AI system efficacy in detecting high-complexity lesions. Moreover, future studies could benefit from a modified and more adaptive methodological framework tailored to the dynamic and continuously evolving landscape of AI applications in dentistry. Such an approach would allow subsequent research to better reflect ongoing technological developments, changing clinical requirements, and emerging standards for the validation of AI-based diagnostic tools.

## 5. Conclusions

Our findings demonstrate that the level of diagnostic agreement between the Diagnocat system and clinical assessment for caries detection in CBCT scans depends on patient gender, age, tooth type, and the individual clinician performing the diagnosis. Notably, the high specificity observed in this study indicates that the system is particularly effective at identifying healthy teeth. This ability to accurately recognize the absence of pathology may enhance its value in screening applications by facilitating the rapid exclusion of cases that do not require intensive diagnostic scrutiny. While the system has significant potential as a diagnostic aid to support the clinical decision-making process, it should not be utilized as a standalone diagnostic tool.

For AI tools to be successfully integrated into routine clinical practice, further clinical studies and interdisciplinary collaboration between AI developers and clinicians are essential.

## Figures and Tables

**Table 1:** Characteristics of the study sample.

Parameter	
Gender	
Women	54 (54%)
Men	46 (46%)
Age [years]	
18–34	31 (31%)
35–54	57 (57%)
≥55	12 (12%)
Number of Patients per Dentist	
Dentist 1	34 (34%)
Dentist 2	33 (33%)
Dentist 3	33 (33%)
Teeth	
Incisors	788 (29%)
Canines	394 (14%)
Premolars	699 (25%)
Molars	874 (32%)

**Table 2:** Overall agreement and errors in Diagnocat analysis compared to clinical evaluation in assessing tooth health.

*n* = 100	Agreement Between Diagnocat and Clinical Evaluation—Healthy Teeth	Missed Detection of Caries by Diagnocat	False Detection of Caries by Diagnocat	Agreement Between Diagnocat and Clinical Evaluation of Caries	Missing Teeth
**Total**	2204	240	64	247	445

**Table 3:** Agreement between Diagnocat analysis and clinical evaluation—detecting caries in women and men.

	Men (*n* = 46)	Women (*n* = 54)
Incisors	30% [8/27]	49% [18/37]
Canines	29% [5/17]	53% [9/17]
Premolars	29% [16/55]	33% [26/78]
Molars	59% [72/122]	69% [93/134]

**Table 4:** Comparison of agreement between Diagnocat analysis and clinical evaluation in caries for all teeth and molars, considering patient gender.

Teeth	Men (*n* = 46)	Women (*n* = 54)	Test Result	*p*-Value	Two-Proportion Z-Test (*p*-Value)
	Agreement Between Diagnocat Analysis and Clinical Evaluatio				
TOTAL	45.7% [101/221]	54.89% [146/266]	4.08 (a)	**0.0435**	**0.0434**
Molars	59.02% [72/122]	69.40% [93/134]	3.01 (a)	0.0829	0.0831

a—Pearson’s chi-square test (χ^2^); bold values indicate statistically significant results (*p* < 0.05); underlined values indicate a trend toward statistical significance (0.05 ≤ *p* < 0.1).

**Table 5:** Agreement between Diagnocat analysis and clinical evaluation by tooth type and patient age group in detecting caries.

	18–34 Years (*n* = 31)	35–54 Years (*n* = 57)	55+ Years (*n* = 12)
Incisors	54% [15/28]	37% [10/27]	11% [1/9]
Canines	53% [8/15]	31% [5/16]	33% [1/3]
Premolars	37% [19/51]	25% [16/65]	41% [7/17]
Molars *	73% [73/100]	58% [80/139]	71% [12/17]

Note: The chi-square test of independence. * Statistically significant difference detected for this tooth type.

**Table 6:** Comparison of agreement between Diagnocat analysis and clinical evaluation by age group and tooth type in detecting caries.

	18–34 Years (*n* = 31)	35–54 Years (*n* = 57)	55+ Years (*n* = 12)	Test Result	*p*-Value	Two-Proportion Z-Test (*p*-Value)		
	Agreement Between Diagnocat Analysis and Clinical Evaluation					1 vs. 2	1 vs. 3	2 vs. 3
TOTAL	59.28% [115/194]	44.94% [111/247]	45.65% [21/46]	9.46 (a)	**0.0088**	**0.0028**	0.0935	NS
Incisors	53.57% [15/28]	37.04% [10/27]	11.11% [1/9]	5.91 (b)	0.0520	NS	**0.0253**	NS
Canines	53.33% [8/15]	31.25% [5/16]	33.33% [1/3]	1.65 (b)	NS	NS	NS	NS
Premolars	37.25% [19/51]	24.62% [16/65]	41.18% [7/17]	2.96 (b)	NS	NS	NS	NS
Molars	73.00% [73/100]	57.55% [80/139]	70.59% [12/17]	6.43 (b)	**0.0401**	**0.0141**	NS	NS

a—Pearson’s chi-square test (χ^2^). b—The chi-square test of independence. NS—Not Significant; bold values indicate statistically significant results (*p* < 0.05); underlined values indicate a trend toward statistical significance (0.05 ≤ *p* < 0.1).

**Table 7:** Comparison of agreement between Diagnocat analysis and clinical evaluation for selected teeth across different age groups in caries detection.

Tooth	18–34 Years (*n* = 31)	35–54 Years (*n* = 57)	55+ Years (*n* = 12)	Test Result *	*p*-Value
	Agreement Between Diagnocat Analysis and Clinical Evaluation				
12	100.0% [3/3]	25.0% [2/8]	33.3% [1/3]	6.30	**0.0428**
15	71.4% [5/7]	33.3% [3/9]	0.0% [0/3]	6.03	**0.0490**
21	62.5% [5/8]	33.3% [1/3]	0.0% [0/3]	4.72	0.0946
28	100.0% [7/7]	70.0% [7/10]	33.3% [1/3]	6.46	**0.0396**
35	20.00% [2/10]	40.0% [4/10]	100.0% [3/3]	7.32	**0.0257**
45	42.9% [3/7]	0.0% [0/8]	0.0% [0/1]	5.88	0.0528
47	83.3% [5/6]	35.7% [5/14]	0.0% [0/1]	5.41	0.0669

* The chi-square test of independence; bold values indicate statistically significant results (*p* < 0.05); underlined values indicate a trend toward statistical significance (0.05 ≤ *p* < 0.1).

**Table 8:** Agreement between Diagnocat analysis and clinical evaluation by dentist—caries detection.

	Dentist 1 (*n* = 34)	Dentist 2 (*n* = 33)	Dentist 3 (*n* = 33)
Incisors	38% [6/16]	53% [10/19]	34% [10/29]
Canines	36% [4/11]	40% [4/10]	46% [6/13]
Premolars	31% [14/45]	30% [10/33]	33% [18/55]
Molars	59% [63/106]	74% [57/77]	62% [45/73]

**Table 9:** Comparison of agreement between Diagnocat analysis and clinical evaluation for different tooth groups by dentist.

	Dentist 1 (*n* = 34)	Dentist 2 (*n* = 33)	Dentist 3 (*n* = 33)	Test Result	*p*-Value	Two-Proportion Z-Test (*p*-Value)		
	Agreement Between Diagnocat Analysis and Clinical Evaluation					1 vs. 2	1 vs. 3	2 vs. 3
TOTAL	48.88% [87/178]	58.27% [81/139]	46.47% [79/170]	4.64 (a)	0.0981	0.0965	NS	**0.0389**
Incisors	37.50% [6/16]	52.63% [6/16]	34.48% [10/29]	1.64 (b)	NS	NS	NS	NS
Canines	36.36% [4/11]	40.00% [4/10]	46.15% [6/13]	0.24 (b)	NS	NS	NS	NS
Premolars	31.11% [14/45]	30.30% [10/33]	32.73% [18/55]	0.06 (a)	NS	NS	NS	NS
Molars	59.43% [63/106]	74.03% [57/77]	61.64% [45/73]	4.49 (a)	NS	**0.0402**	NS	NS

a—Pearson’s chi-square test (χ^2^). b—The chi-square test of independence. NS—Not Significant; bold values indicate statistically significant results (*p* < 0.05); underlined values indicate a trend toward statistical significance (0.05 ≤ *p* < 0.1).

**Table 10:** Comparison of agreement between Diagnocat analysis and clinical evaluation for individual teeth by dentist.

Tooth	Dentist 1	Dentist 2	Dentist 3	Test Result *	*p*-Value
	Agreement Between Diagnocat Analysis and Clinical Evaluation				
15	33.3% [3/8]	100.0% [3/3]	28.6% [2/7]	6.03	**0.0490**
16	71.4% [5/7]	100.0% [6/6]	50.0% [4/8]	5.66	0.0590
23	50.0% [3/6]	0.0% [0/3]	75.0% [3/4]	5.13	0.0770
47	22.2% [2/9]	57.1% [4/7]	80.0% [4/5]	4.97	0.0835

* The chi-square test of independence; bold values indicate statistically significant results (*p* < 0.05); underlined values indicate a trend toward statistical significance (0.05 ≤ *p* < 0.1).

**Table 11:** Age distribution of patients by dentist—bivariate analysis.

Age	Summary Bivariate Table: Observed Frequencies			
	Dentist 1	Dentist 2	Dentist 3	Total
18–34	8	13	10	31
% total	8%	13%	10%	31%
35–54	20	19	18	57
% total	20%	19%	18%	57%
55+	6	1	5	12
% total	6%	1%	5%	12%
Total	34	33	33	100
% total	34%	33%	33%	100%

Note: χ^2^ = 4.813137; *p* = 0.30701.

**Table 12:** Gender distribution of patients by dentist—bivariate analysis.

Gender	Summary Bivariate Table: Observed Frequencies			
	Dentist 1	Dentist 2	Dentist 3	Total
Women	18	17	19	54
% total	18%	17%	19%	54%
Men	16	16	14	46
% total	16%	16%	14%	46%
Total	34	33	33	100
% total	34%	33%	33%	100%

Note: χ^2^ = 0.267; *p* = 0.875.

## Data Availability

Data are available on request due to privacy/ethical restrictions.
